# Disease characteristics and serological responses in patients with differing severity of COVID-19 infection: A longitudinal cohort study in Dhaka, Bangladesh

**DOI:** 10.1371/journal.pntd.0010102

**Published:** 2022-01-04

**Authors:** Afroza Akter, Tasnuva Ahmed, Imam Tauheed, Marjahan Akhtar, Sadia Isfat Ara Rahman, Fatema Khaton, Faisal Ahmmed, Jannatul Ferdous, Mokibul Hassan Afrad, Zannat Kawser, Mohabbat Hossain, Rabeya Khondaker, Mohammad Abul Hasnat, Mostafa Aziz Sumon, Asif Rashed, Shuvro Ghosh, Stephen B. Calderwood, Richelle C. Charles, Edward T. Ryan, Purvesh Khatri, Holden Terry Maecker, Gerlinde Obermoser, Bali Pulendran, John D. Clemens, Sayera Banu, Tahmina Shirin, Regina C. LaRocque, Jason B. Harris, Taufiqur Rahman Bhuiyan, Fahima Chowdhury, Firdausi Qadri

**Affiliations:** 1 International Centre for Diarrhoeal Disease Research Bangladesh (icddr,b), Dhaka, Bangladesh); 2 Institute for Developing Science & Health Initiatives (ideSHi), Dhaka, Bangladesh; 3 Kurmitola General Hospital, Dhaka, Bangladesh; 4 Mugda Medical College & Hospital, Dhaka, Bangladesh; 5 Division of Infectious Diseases, Massachusetts General Hospital, Boston, Massachusetts, United States of America; 6 Departments of Medicine and Pediatrics, Harvard Medical School, Boston, Massachusetts, United States of America; 7 Department of Immunology and Infectious Diseases, Harvard T.H. Chan School of Public Health, Boston, Massachusetts, United States of America; 8 Stanford University, Stanford, California, United States of America; 9 UCLA Fielding School of Public Health, Los Angeles, California, United States of America (JD Clemens MD); 10 Korea University School of Medicine, Seoul, South Korea (JD Clemens MD); 11 Institute of Epidemiology, Disease Control and Research, Dhaka, Bangladesh; Minia University, EGYPT

## Abstract

**Background:**

COVID-19 caused by SARS-CoV-2 ranges from asymptomatic to severe disease and can cause fatal and devastating outcome in many cases. In this study, we have compared the clinical, biochemical and immunological parameters across the different disease spectrum of COVID-19 in Bangladeshi patients.

**Methodology/Principal findings:**

This longitudinal study was conducted in two COVID-19 hospitals and also around the community in Dhaka city in Bangladesh between November 2020 to March 2021. A total of 100 patients with COVID-19 infection were enrolled and classified into asymptomatic, mild, moderate and severe cases (n = 25/group). In addition, thirty age and sex matched healthy participants were enrolled and 21 were analyzed as controls based on exclusion criteria. After enrollment (study day1), follow-up visits were conducted on day 7, 14 and 28 for the cases.

Older age, male gender and co-morbid conditions were the risk factors for severe COVID-19 disease. Those with moderate and severe cases of infection had low lymphocyte counts, high neutrophil counts along with a higher neutrophil-lymphocyte ratio (NLR) at enrollment; this decreased to normal range within 42 days after the onset of symptom. At enrollment, D-dimer, CRP and ferritin levels were elevated among moderate and severe cases. The mild, moderate, and severe cases were seropositive for IgG antibody by day 14 after enrollment. Moderate and severe cases showed significantly higher IgM and IgG levels of antibodies to SARS-CoV-2 compared to mild and asymptomatic cases.

**Conclusion/Significance:**

We report on the clinical, biochemical, and hematological parameters associated with the different severity of COVID-19 infection. We also show different profile of antibody response against SARS-CoV-2 in relation to disease severity, especially in those with moderate and severe disease manifestations compared to the mild and asymptomatic infection.

## Author summary

In Bangladesh, very little is known about disease characteristics and serological responses in different spectrum of COVID-19 disease in patients. The risk factors responsible for causing severe COVID-19 illness, as well as data on clinical, biochemical and immunological parameters is still lacking or insufficient across different geographical regions for the prediction of the disease outcomes. This is the first longitudinal study in Bangladesh to evaluate the data on clinical, hematological parameters, viral load and antibody responses in COVID-19 infected asymptomatic, mild, moderate, and severe patients. Our study showed that older age, male gender, multiple co-morbid conditions and elevated NLR, D-dimer, ferritin and CRP levels can help clinicians to predict progression to more severe outcomes. Patients with moderate and severe disease developed higher levels of IgM and IgG antibodies to the receptor binding domain (RBD) of the spike protein of SARS-CoV-2 compared to patients suffering from mild disease or those with asymptomatic infections. However, all infected individuals developed antibody responses that were higher than that seen in healthy controls.

## Introduction

The COVID-19 pandemic caused by Severe Acute Respiratory Syndrome-Coronavirus-2 (SARS-CoV-2) emerged in late 2019 in China and has rapidly spread globally [1]. Information on the risk factors responsible for causing severe illness, as well as clinical and laboratory parameters that predict more severe disease, across different geographical regions is still lacking. Since March 2020, SARS-CoV-2 has led to widespread transmission of COVID-19 in Bangladesh [1,2]. The prevalence of COVID-19 has been high in Dhaka city, which is the epicenter of the infection [3]. At present, about 782,129 cases and 12,211 deaths have been reported in the country. Average infectivity rate in Bangladesh is about 7.5% with 1.56% mortality rate which is lower than seen in the USA, Brazil, UK, France, South Africa, Philippines and Pakistan [4]. In Bangladesh, 80% of infected people suffer from mild disease or are asymptomatic [3]. The remaining suffer from moderate to severe disease, with about 5–9% of the hospitalized patients requiring intensive care unit admission [5].

The severity of COVID-19 infection is related to co-morbid conditions, alteration of immune response and lack of immunity to SARS-CoV-2 [6]. The viral load in nasopharyngeal swab specimens (NPS) is also associated with severity [7]. The current study was designed to compare the clinical, biochemical, and hematological parameters, viral load, and antibody responses in the COVID-19 patients with different grades of disease presentation (asymptomatic, mild, moderate, and severe) compared to the healthy controls. We also explored the association of initial laboratory and clinical parameters with the immunologic responses in the cohort at one month of follow-up.

## Methods

### Ethics statement

This study was approved by the Institutional Review Board of International Centre for Diarrhoeal Disease Research (icddr,b) and the Directorate General of Health Services (DGHS) of Bangladesh. Informed written consent was obtained from all participants according to the ‘Declaration of Helsinki’ regulation and guidelines.

### Participants and study sites

We report on the longitudinal cohort study, conducted in Dhaka, Bangladesh between November, 2020 to March, 2021. We enrolled 100 patients who were SARS-CoV-2 reverse transcription polymerase chain reaction (RT-PCR) test positive (≥18 years of age) who were categorized as asymptomatic, mild, moderate, and severe cases (n = 25; per group) and compared them with 30 age and sex matched healthy controls and 21 were analyzed as controls. Nine participants were excluded from the analysis due to SARS-CoV-2 seropositivity at baseline. These controls were judged healthy by medical personnel, had no history of COVID-19, were RT-PCR negative for SARS-CoV-2 during enrollment and had no clinical signs and symptoms in the two weeks prior to enrollment. We classified the disease severity of the patients based on clinical symptoms and oxygen saturation (SpO_2_) according to the WHO guidelines [8], which were assessed from the hospital admission records or the patient’s clinical condition during enrollment. Respiratory distress with SpO_2_<90% was considered as severe whereas respiratory distress with SpO_2_ ≥90% was moderate. The mild cases did not have respiratory distress but could have other symptoms. Asymptomatic cases were defined as RT-PCR positive (cycle threshold less than 40) having no sign or symptom during enrollment [9].

Patients were enrolled from designated COVID-19 hospitals, Mugda Medical College and Hospital and Kurmitola General Hospital, as well as from the community (non-hospitalized patients) in Dhaka city. Follow-up of participants were continued in these two hospitals and in the Mirpur field site.

### Collection of clinical and laboratory data

Socio-demographic data, clinical features, vital signs, co-morbidities, exposure or contact history with COVID-19 patients and visit to a health care facility 14 days prior to diagnosis were recorded. Patients were enrolled on day 1 and prospectively followed up at day 7, 14 and 28. The date of first symptom for symptomatic cases was considered as the date of onset and date of exposure with any COVID-19 patient or RT-PCR positive report was considered as the date of onset for asymptomatic cases.

We collected NPS and blood from all patients on all study days. Clinical laboratory investigation consisting of complete blood count (CBC), random blood sugar (RBS), alanine aminotransferase, aspartate aminotransferase, creatinine, C-reactive protein (CRP), lactate dehydrogenase (LDH), ferritin, creatine kinase (CK) and D-dimer were measured on study day 1 and day 28, while procalcitonin was only measured on day 1 as these tests are usually performed for COVID-19 patients according to national guideline of management of COVID-19 patients in Bangladesh [10]. The NLR value was measured by the ratio of absolute neutrophil count to absolute lymphocyte count to explore the relatedness to disease severity [11]. Antibody responses to the receptor binding domain (RBD) of the spike protein of SARS-CoV-2 (IgM and IgG) were measured on different study days. Specimens from healthy controls were collected only once at enrolment. Analyses were carried out at the icddr,b and the Institute for Developing Science and Health Initiatives (ideSHi) laboratories.

### SARS-CoV-2 RT-PCR and viral load quantification from NPS

Viral RNA was extracted using the magnetic bead based Nexor 32 Fully Automated Nucleic Acid Extractor (Nucleic Acid Extraction or Purification Kit, Beijing Lepu Medical Technology Co., Ltd, China). RT-PCR and estimated viral load were quantified using the Chinese CDC 2019-nCoV_N2 primers and probe set [12]. Briefly, RT-PCR was performed in 20 μl reaction volumes and each reaction contained 5 ul template RNA, 10 μl of iTaq Universal Probes Reaction Master Mix (Biorad, CA, USA), 0.5 ul of iScript Reverse Transcriptase, 500 nM of both forward and reverse primer and 125 nM of probe. For the RT-PCR test, a positive test was set at a threshold cycle (Ct value) of less than 40. The Human RNase P gene was used as an internal control to monitor viral RNA extraction efficiency [12]. Viral copy numbers were quantified using N2 quantitative PCR standards in 8-fold dilutions to generate a standard curve [13]. The assay was run in duplicates for each specimen.

### RBD-specific antibody responses using enzyme linked immunosorbent assay (ELISA)

We determined the IgM and IgG antibody response specific to RBD in sera collected from patients and healthy controls [1,14]. The RBD-specific antibody concentrations (ng/mL) were measured using isotype-specific anti-RBD monoclonal antibodies (Mab CR3022) using previously described methods [1].

To determine the antibody concentration in serum, CR3022 was used to generate a standard curve (starting concentration 25 ng/ml) for both IgG and IgM. We have previously validated this assay using serum specimens (n = 100) collected from RT-PCR positive COVID-19 patients, pre-pandemic healthy controls (frozen sera specimens that had been collected between 2015–2018 and stored in the freezer archive), influenza infected patients and serum collected from surveillance of Japanese encephalitis [1] and compared the responses with commercially available ELISA kits (Euroimmun, Germany; Wantai, China). To determine the cut-off for seropositivity, the median plus the range of concentrations of SARS-CoV-2 IgG and IgM antibodies measured in pre-pandemic serum samples were used. Based on this, 500 ng/ml (0.5 μg/ml) was set as the positive cut-off value for both IgG and IgM antibodies [14].

### Statistical analysis

The demographic and clinical characteristics were stratified by severity and measured as mean with 95% confidence interval (CI) for continuous data, median with inter-quartile range (IQR) for ordinal data and percentage for categorical data. For the comparison of clinical laboratory values, we calculated geometric mean (GM) with 95% CI by disease severity. If two CI did not overlap, it was assumed that the point estimates and GMs were significantly different from each other. Scatter plots with predicted smoothing lines were created to see the changes of clinical laboratory values over time after disease onset. Spline regression method was used to smooth the predicted lines with three polynomial segments. Comparison of antibody responses (IgG and IgM) between symptomatic cases (mild, moderate and severe), asymptomatic cases and healthy controls were analyzed by the Mann-Whitney U test [1]. All analyses were carried out using R-statistical software (“ggplot2” package for the scatter diagram, and “dplyr” package for data) and GraphPad Prism 6.0.

## Results

### Clinical and demographic features

**Fig 1:**
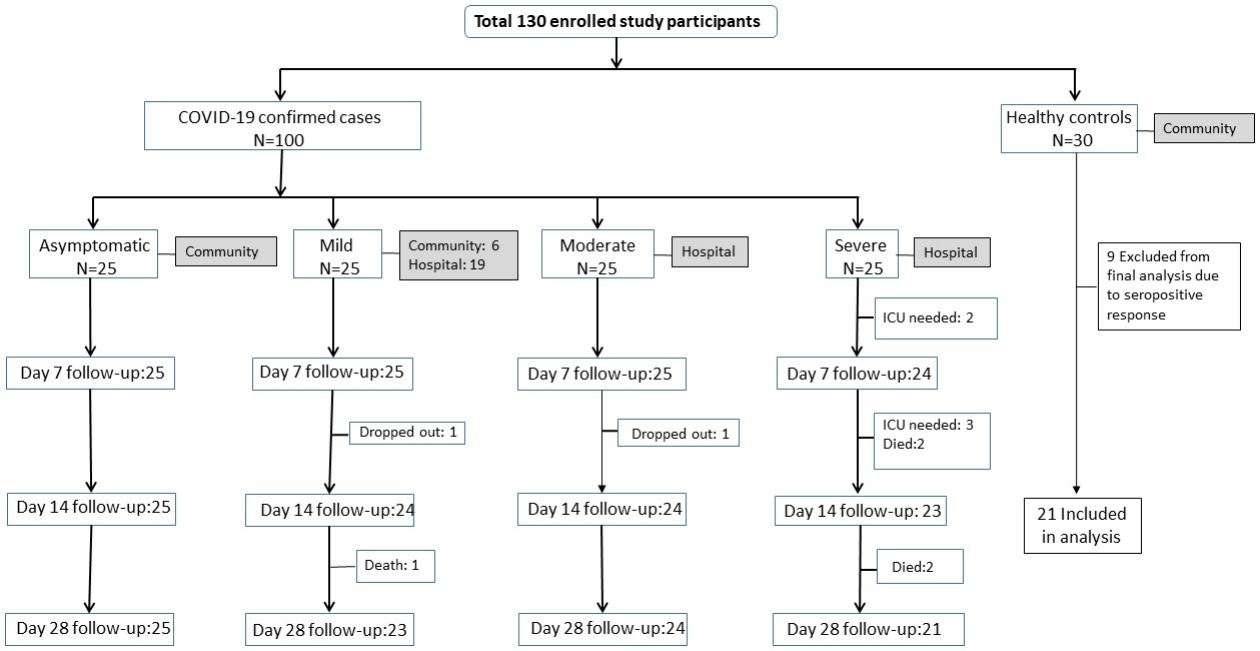
Enrollment Diagram.

Of 100 cases enrolled, seven were lost to follow-up by day 28 including death (Fig 1). Among the cases, 60% were male and median age was 47 years. The mean age for asymptomatic cases was 37.8 years (95% CI: 32.7–42.8) while the mean age for severe cases was 55 years (95% CI: 50.6–60.4). Thirty healthy controls were enrolled, of whom 9 were excluded from the analysis due to SARS-CoV-2 seropositivity. About 68% of symptomatic cases were male, where 72% (n = 18) of those suffered from moderate and 68% (n = 17) from severe disease. Severe cases had higher BMI (27.4 kg/m^2^) compared to the others. About 56% of the mild cases had an exposure history to COVID-19 (Table 1).

**Table 1:** Socio-demographic characteristics of COVID-19 patients and healthy controls.

Variables	COVID-19 negative (Controls) n = 21	COVID-19 positive patients			
		Asymptomatic n = 25	Mild n = 25	Moderate n = 25	Severe n = 25
Age (years)*	42.2 (36.7–47.7)	37.8 (32.7–42.8)	44.7 (38.9–40.4)	49.4 (44.3–54.4)	55.5 (50.6–60.4)
Sex: Male, n (%)	12 (57)	9 (36)	16 (64)	18 (72)	17 (68)
BMI (kg/m^2^)*	25.4 (23.7–27.1)	24.4 (23.1–25.7)	26.1 (24.9–27.2)	25.5 (24.2–26.8)	27.4 (25.5–29.4)
Education ≤High school, n (%)	7 (33.3)	10 (40)	3 (12)	12 (48)	12 (48)
Average Household Monthly Income (BDT) **	50000 (36000,100000)	50000 (50000,100000)	60000 (50000,100000)	50000 (25000,100000)	50000 (40000,100000)
COVID-19 positive family members* ^a^	-	2.4 (1.8–2.9)	1.6 (1.0–2.3)	1.0 (0.4–1.5)	1.2 (0.6–1.7)
Exposure history, n (%)^b^					
Contact with confirmed case	-	11 (44)	14 (56)	8 (32)	4 (16)
Visit to health care facility	-	3 (12)	14 (56)	6 (24)	6 (24)

* Values are mean with 95% confidence interval (CI);

** Data values presented as median (inter quartile range, IQR)

^a^ No. of family members diagnosed with COVID-19 during the disease period of the participant.

^b^ Exposure history of cases in 14 days prior to diagnosis of COVID-19

Duration between symptom onset and enrollment was less in mild cases than moderate and severe cases. The median days between symptom onset and diagnosis by RT-PCR were 5, 5, and 6 days for mild, moderate, and severe cases, respectively. About 72% symptomatic cases reported fever as the first symptom at disease onset. The moderate and severe cases reported shortness of breath, fever and cough on enrollment (85–100%), while mild cases more commonly had fever (92%), loss of taste (60%) and cough (52%). Gastrointestinal (nausea, vomiting and diarrhea) and other systemic symptoms were least reported symptoms (0–20%). The mean oxygen saturation (SpO_2_) recorded during hospitalization was 97.7%, 94.0% and 82.6% among the mild, moderate, and severe cases, respectively (Table 2).

**Table 2:** Clinical characteristics of symptomatic COVID-19 patients with different severities at enrollment.

Variables	Mild n = 25	Moderate n = 25	Severe n = 25
Interval period^a^			
Duration between symptom onset and enrollment	9 (6,10)	10 (9,12)	11 (9,13)
Duration between symptom onset and diagnosis	5 (2,6)	5 (4,6)	6 (3,8)
Duration between symptom onset and hospitalization	6 (4,9)	7 (6,9)	8 (6,10)
First symptom at disease onset, n (%)			
Cough	1 (4)	3 (12)	5 (20)
Fever	19 (76)	17 (68)	18 (72)
Shortness of breath	-	1 (4)	-
Headache	2 (8)	1 (4)	-
Loss of smell	1 (4)	-	-
Loss of Taste	1 (4)	-	1 (4)
Myalgia	-	1 (4)	-
Runny nose	1 (4)	1 (4)	1 (4)
Sneezing	-	1 (4)	-
Enrollment (day 1) symptoms, n (%)			
Fever	23 (92)	22 (88)	25 (100)
Cough	13 (52)	21 (84)	22 (88)
Sore throat	3 (12)	1 (4)	3 (12)
Shortness of breath	0	25 (100)	25 (100)
Loss of smell	11 (44)	6 (24)	6 (24)
Loss of taste	15 (60)	9 (36)	9 (36)
Runny nose	4 (16)	4 (16)	1 (4)
Chest pain	2 (8)	2 (8)	1 (4)
Muscle aches	3 (12)	4 (16)	5 (20)
Joint pain	4 (16)	5 (20)	1 (4)
Fatigue	3 (12)	-	-
Malaise	1 (4)	-	-
Headache	8 (32)	7 (28)	2 (8)
Nausea	1 (4)	-	-
Vomiting	3 (12)	1 (4)	2 (8)
Diarrhea	2 (8)	5 (20)	3 (12)
Other^§^	2 (8)	1 (4)	2 (8)
Highest recorded temperature (°F), mean (CI)^b^	101.6 (101.1–102.1)	102.2 (101.6–102.7)	101.8 (101.4–102.2)
SpO_2_ on admission (%),mean (CI)^c^	97.7 (97.0–98.4)*	94.0 (92.9–95.2)**	**82.6 (77.8–87.3)** **

Asymptomatic and healthy control participants are not presented here as they presented with no symptoms or did not have hospitalization information. Bold values indicate significant difference among comparison groups.

^a^ Data is presented as median days (interquartile range, IQR);

^b^ Patients who complained of fever only;

^c^ Only for hospitalized patients;

^§^ Back pain (2 severe cases), generalized weakness (1 mild case), hiccup (1 mild case), sneezing (1 moderate case), tonsillitis (1 mild case).

*19 out of 25 patients were hospitalized as they were unable to isolate at home;

**All the patients were hospitalized.

Moderate and severe cases had higher systolic blood pressure and respiratory rate than mild and asymptomatic cases. About 84% of asymptomatic cases had no co-morbid diseases, while 52% of the severe cases had more than one co-morbid disease. The majority of the severe cases reported diabetes (60%) followed by hypertension (36%), and asthma (32%) (Table 3).

**Table 3:** Vital signs and distribution of co-morbidities among different severities of COVID-19 patients.

Variables	Asymptomatic (n = 25)	Mild (n = 25)	Moderate (n = 25)	Severe (n = 25)
Respiratory rate^a^	19.1 (17.9–20.2)	20.6 (19.1–22.2)	**27.8 (25.1–30.5)**	**32.3 (29.8–34.9)**
Pulse rate^a^	83.8 (78.7–89.1)	84.4 (78.2–90.6)	91.1 (84.8–97.4)	84.6 (79.5–89.6)
Systolic Pressure(mmHg)^a^	117.2 (111.2–123.2)	121.8 (116.0–127.6)	128.2 (121.4–134.9)	127 (118.3–135.6)
Diastolic Pressure(mmHg)^a^	76.1 (71.4–80.8)	79.6 (76.1–83.1)	81.2 (76.6–85.8)	74.7 (69.6–79.8)
No comorbidity	21 (84)	12 (48)	9 (36)	5 (20)
1 comorbidity	3 (12)	8 (32)	9 (36)	7 (28)
>1 comorbidity	1 (4)	5 (20)	7 (28)	13 (52)
Diabetes	1 (4)	4 (16)	8 (32)	15 (60)
Heart disease	-	2 (8)	4 (16)	2 (8)
Hypertension	3 (12)	6 (24)	8 (32)	9 (36)
Asthma	-	4 (16)	3 (12)	8 (32)
Tuberculosis	-	-	-	2 (8)
Liver disease	-	-	1 (4)	-
Kidney disease	1 (4)	1 (4)	1 (4)	1 (4)
Other^§^	1 (4)	1 (4)	-	-

Bold values indicate significant difference among comparison groups. Comorbidity data presented as n (%)

^a^ Values given as mean (95% confidence interval, CI).

^§^ Other comorbidities included hypothyroidism and epilepsy.

### Laboratory features associated with disease severity

**Fig 2:**
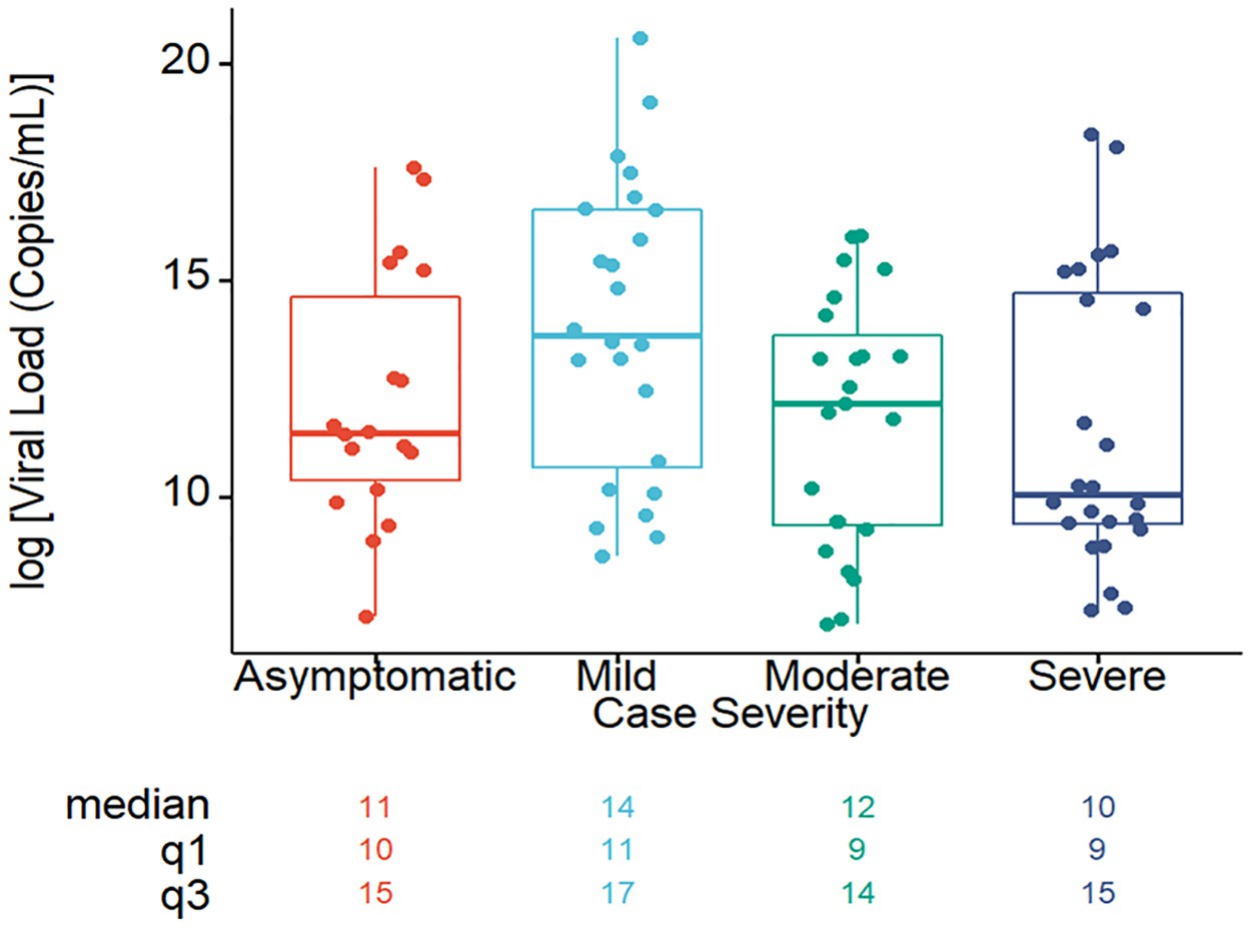
Viral load of COVID-19 patients with different severities during the study period. The SARS-CoV-2 viral load observed among patients with different severities on study day 0. The horizontal line inside each box represents the median value of the viral load copies/mL with interquartile ranges. The dots represent cases.

The viral load was higher in mild cases compared to the others (Fig 2). The median value of viral load in asymptomatic, mild, moderate and severe cases was found to be 11(IQR: 10, 15), 14(IQR: 11, 17), 12(IQR: 9, 14) and 10 (IQR: 9, 15) respectively.

**Fig 3:**
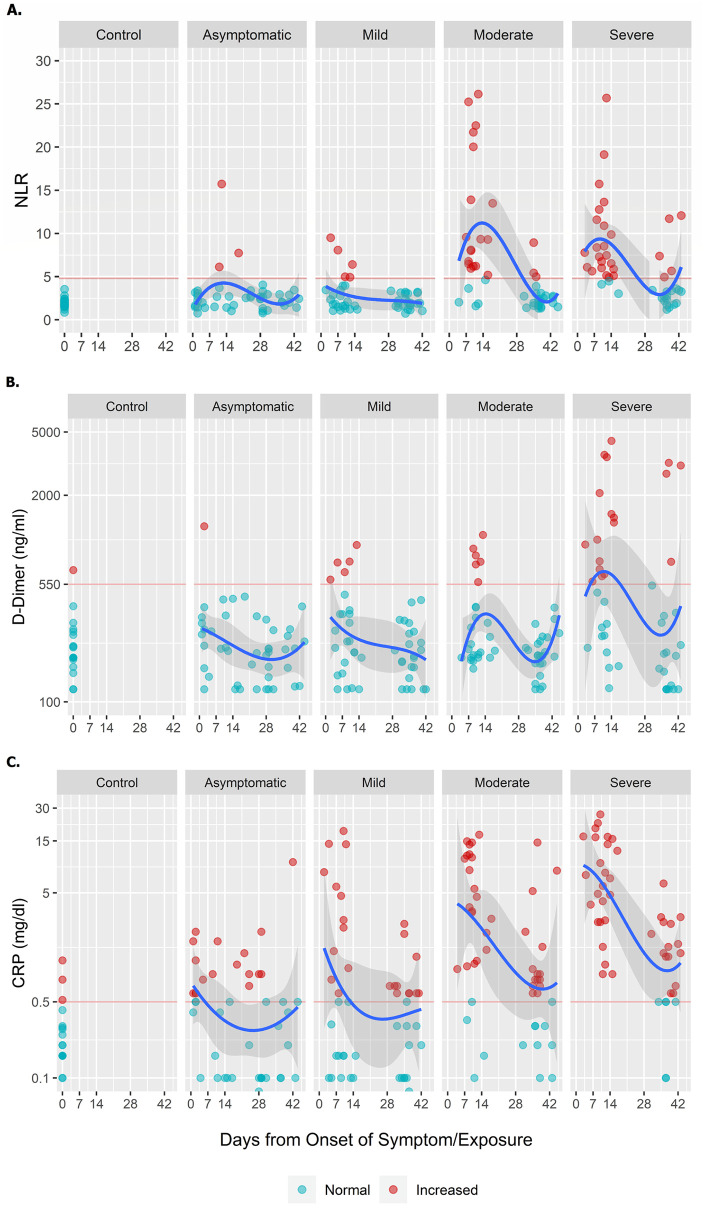
Trend of NLR, D-dimer and CRP among cases and controls observed over the follow-up period. All values for the symptomatic cases are measured from the date of symptom onset. Follow-up duration for asymptomatic cases are measured from the date of exposure. Healthy control participants provided samples only at one time point and values have been reported as day 0. Cut-off value for NLR was 4.795, D-Dimer is 550ng/ml and CRP is 0.5mg/dl. The blue line represents mean data over the follow-up period. Grey shaded area shows 95% CI of the mean values. Red horizontal lines reflect the normal reference values.

The clinical, hematological, and biochemical findings for the cases and healthy controls on day 1 and 28 are summarized in Table 4. An increased neutrophil count was observed among the moderate (80.0%) and severe (80.6%) cases when compared to the other patients. Moderate and severe cases had significantly decreased lymphocyte counts, with a GM of 9.2% and 10.5%, compared to the asymptomatic and mild cases. Decreased lymphocyte percentage persisted through day 28 in the severe COVID-19 group (18.2%). Majority of the moderate and severe cases had an elevated NLR within the first ten days of disease onset, which eventually decreased to normal within 42 days from disease onset (Fig 3A).

**Table 4:** Laboratory parameters in COVID-19 patients with differing severities.

Parameters *Normal reference*	Day	Control	Asymptomatic	Mild	Moderate	Severe
TLC (10^9^/L) *4*.*0–11*.*0*	1	6.6 (5.9–7.4)	5.8 (5.1–6.7)	5.5 (5–6.1)	8.2 (7.1–9.4)	7.4 (6.5–8.5)
	28	-	6.2 (5.5–6.9)	5.8 (5.4–6.3)	5.3 (4.8–5.9)	6.1 (5–7.4)
Diff. Neut (%) *40–75*	1	56.4 (53.3–59.8)	56.7 (52–61.7)	61.6 (57.2–66.3)	**80 (75.2–85.2)**	**80.6 (77.7–83.5)**
	28	-	55.5 (51.7–59.5)	54.3 (51.1–57.7)	55.9 (52.1–60.1)	59.5 (53.8–65.8)
Diff. Lymph (%) *20–45*	1	30.2 (27.7–33)	22.9 (19.1–27.3)	24.1 (20.1–28.9)	**9.2 (6.9–12.2)**	**10.5 (9–12.3)**
	28	-	25.8 (22.7–29.3)	26.2 (23.8–28.8)	23.4 (20.6–26.6)	18.2 (15.2–21.8)
Abs.Neut (10^9^/L) *2*.*0–7*.*5*	1	3.7 (3.2–4.3)	3.3 (2.7–4)	3.4 (3–3.9)	**6.6 (5.5–7.8)**	**6 (5.1–7)**
	28	-	3.4 (2.9–4)	3 (2.5–3.5)	3 (2.6–3.5)	3.7 (2.8–4.8)
Abs. Lymph (10^9^/L) *1*.*5–4*.*0*	1	2 (1.7–2.3)	1.3 (1.1–1.6)	1.3 (1.1–1.6)	**0.7 (0.6–1)**	**0.8 (0.7–0.9)**
	28	-	1.5 (1.4–1.7)	1.5 (1.4–1.7)	1.2 (1.1–1.4)	**1.1 (1–1.3)**
Abs. Mono (10^9^/L) *0*.*2–0*.*8*	1	0.4 (0.3–0.5)	0.8 (0.6–0.9)	0.4 (0.3–0.5)	0.5 (0.4–0.6)	0.5 (0.4–0.6)
	28	-	0.8 (0.6–0.9)	0.8 (0.7–0.9)	0.7 (0.6–0.9)	0.8 (0.7–1)
Plt (10^9^/L) *150–450*	1	260.8 (223.6–304.2)	230.6 (203.4–261.4)	219.9 (191.2–253)	239.7 (208.1–276)	287.8 (255.8–323.9)
	28	-	200.1 (137.6–290.9)	225.1 (192.1–263.7)	217.1 (191.8–245.7)	202.4 (174.8–234.4)
RBS (mmol/L) *4*.*20–7*.*80*	1	6.1 (5.5–6.7)	5.8 (5–6.7)	7.2 (6.1–8.6)	**11.7 (9.7–14.1)**	**10.8 (9.1–12.8)**
	28	-	5.1 (4.2–6.2)	5.9 (4.4–7.9)	8.9 (7.3–10.7)	9.7 (7.9–12)
Cr (μmol/L) *64–104*	1	76.2 (68.8–84.5)	71.1 (64–79)	77.6 (69.3–87)	85.4 (74–98.6)	80.5 (72.2–89.7)
	28	-	71.3 (59.6–85.3)	87.1 (69.2–109.6)	83.4 (71.8–96.8)	74.5 (65.7–84.6)
ALT (U/L) *<50*	1	28.8 (21.4–38.8)	29.8 (23.6–37.5)	41.2 (31.2–54.2)	56.9 (39.4–82.3)	61 (43.5–85.7)
	28	-	27.6 (21.8–34.9)	34.6 (27.8–43.1)	50.5 (39–65.4)	39.9 (33.6–47.3)
AST (U/L) *<50*	1	25.9 (22.4–30)	28.2 (24–33)	39.3 (30.8–50.1)	48.1 (38.1–60.7)	48.6 (38.1–62.2)
	28	-	27.3 (24–30.9)	26.4 (22.9–30.6)	31.6 (26.7–37.5)	28.4 (23.8–33.8)
LDH (U/L) *<248*	1	193 (182.8–203.8)	202.9 (180.2–228.6)	240.4 (208.5–277.1)	290.8 (229–369.3)	**418.2 (367.1–476.5)**
	28	-	189.9 (175.7–205.3)	188.3 (162.5–218.2)	241.5 (215.7–270.3)	**268.8 (234.6–308.1)**
CK (U/L) *<171*	1	112.3 (96.1–131.1)	101.6 (73.7–140)	121.8 (86.1–172.2)	85.6 (52–140.7)	109.9 (67.6–178.9)
	28	-	116.2 (85.9–157.2)	88.4 (56.6–138)	60.6 (51.8–71)	56.4 (42.3–75.1)
CRP (mg/dL) *<0*.*5*	1	0.2 (0.2–0.3)	0.5 (0.3–0.7)	0.7 (0.4–1.5)	**2.8 (1.6–5)**	**6 (4–9)**
	28	-	0	0	**0.7 (0.4–1.1)**	**1 (0.6–1.6)**
Ferritin (ng/mL) *≤274*.*66*	1	43.9 (25.9–74.2)	40.8 (23–72.6)	197.6 (124.1–314.6)	448.8 (294.9–683)	460.6 (287.4–737.9)
	28	-	30.9 (16.4–58.1)	76 (49.8–115.8)	124.9 (74.4–209.7)	159.5 (95.8–265.5)
D-Dimer (ng/mL) *<550*	1	217.2 (183.9–256.6)	246.2 (197.3–307.1)	278.4 (217.1–357.1)	310.7 (245–394)	**625.4 (418.5–934.4)**
	28	-	207.8 (175.3–246.4)	203.7 (172.5–240.5)	201.3 (177.5–228.3)	295.7 (184.5–474.1)
PCT (ng/mL) *<0*.*1*	1	0 (0–0)	0.1 (0.1–0.1)	0.1 (0–0.1)	0.1 (0.1–0.2)	0.1 (0.1–0.1)

Data displayed as GM (95% CI);

Bold values indicate significant differences among comparison groups.

Abbreviation: Total leucocyte count (TLC), Differential neutrophil count (Diff. Neut), Differential lymphocyte count (Diff. Lymph), Absolute neutrophil count (Abs.Neut), Absolute lymphocyte count (Abs.Lymph), Absolute monocyte count (Abs. Mono), Platelet (Plt) Random blood sugar (RBS), Alanine aminotransferase (ALT), Aspartate aminotransferase (AST), Creatinine (Cr), C-reactive protein (CRP), Lactate dehydrogenase (LDH), Creatine Kinase (CK) and Procalcitonin (PCT).

Elevated RBS (>7.8 mmol/L) were observed in patients with moderate (GM 11.7) and severe (GM 10.8) disease (Table 4). Of the 100 cases, 28% had diabetes and 48% presented with elevated RBS on study day 1.

The inflammatory markers, LDH, CRP, and ferritin were significantly elevated on study day 1 among moderate and severe cases compared to other groups. Severe cases had elevated levels of D-dimer (625.4 ng/mL) on day 1 compared to other groups (Table 4, Fig 3B). The LDH in severe cases remained elevated on study day 28 (268.8) compared to other groups. The CRP among both moderate (0.7) and severe (1.0) cases remained elevated at day 28 when compared to mild or asymptomatic cases (Table 4, Fig 3C).

### SARS-CoV-2 specific antibody responses

**Fig 4:**
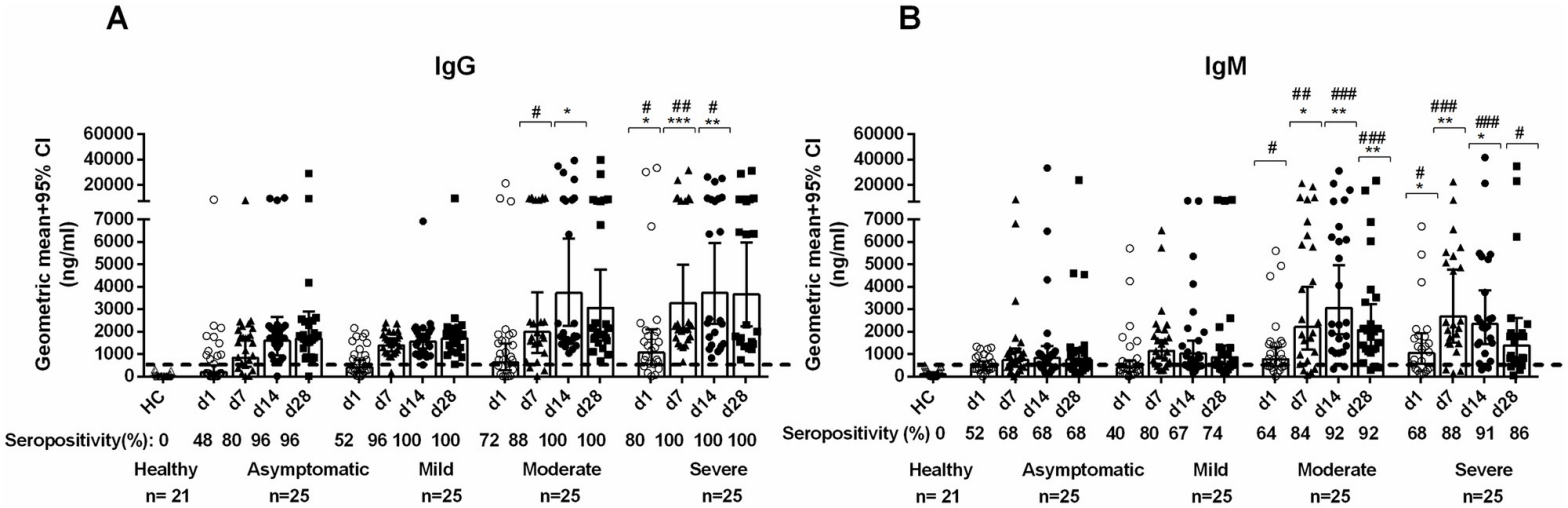
SARS-CoV-2 specific antibody responses in COVID-19 cases and controls. The SARS-COV-2 RBD specific serum (A) IgG and (B) IgM antibody concentrations in healthy individuals (n = 21) and asymptomatic (n = 25), mild (n = 25), moderate (n = 25) and severe (n = 25) COVID-19 patients on study day 1, day 7, day 14 and day 28. Bars represent geometric mean concentration with 95% CI and the dotted line indicates the cut-off (500 ng/ml) limit of seropositivity. Compared to healthy controls, all patients had significantly higher (P<0.0001) IgM and IgG antibodies at all time points. Asterisks (*) represent significant differences between mild patients vs. moderate and severe patients and hashes (#) represent significant differences between asymptomatic patients vs. moderate and severe patients on different days. Statistical analysis was performed using the Mann-Whitney test. *^/#^*P* <0.05, **^/##^*P*<0.01, ***^/###^*P* <0.001.

The RBD-specific IgG and IgM antibody responses in the different groups of COVID-19 patients were higher on all study days when compared to controls (P<0.0001). Patients with asymptomatic or mild disease had less prominent IgG and IgM responses to RBD compared to those with moderate or severe disease. More than 96% of patients with all disease severities became seropositive for IgG by day 14, and these remained elevated for over a month (Fig 4).

### Correlation of NLR, D-dimer and CRP on day 1 with SARS-CoV-2 specific antibody responses on study day 28

**Fig 5:**
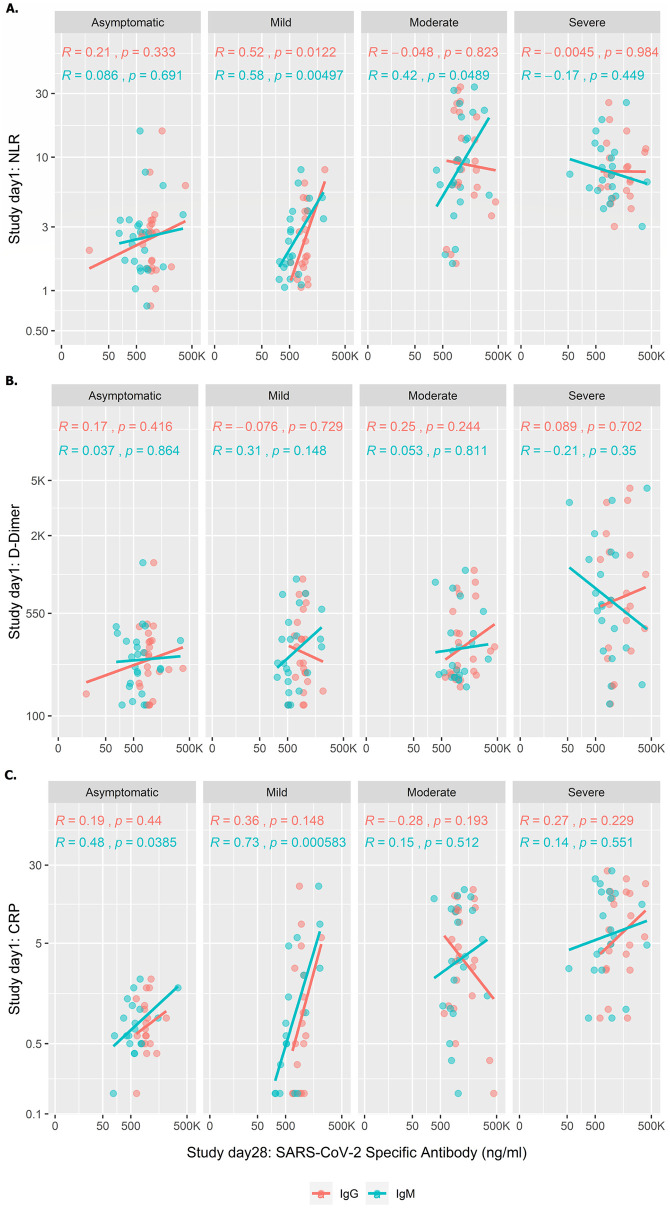
Correlations of early NLR, D-dimer and CRP values with SARS-CoV-2 specific antibody responses at 1 month. NLR, D-Dimer and CRP values from day 1 were correlated with day 28 IgG and IgM antibody responses to explore the correlation of early inflammation with subsequent immune responses among patients with different disease severity. Straight lines created based on logarithm of X- axis and presented in an anti-logarithm scale.

We carried out correlation analyses of baseline laboratory parameters (NLR, D-dimer and CRP) with SARS-CoV-2 specific antibodies (IgG and IgM) at day 28 (Fig 5). Moderate and severe patients had increased NLR and CRP on enrollment compared to mild and asymptomatic patients and also had higher IgM and IgG concentration later in illness compared to these groups.

However, no significant correlation was seen in the level of inflammatory markers on day 1 in patients with moderate and severe illness with the subsequent level of immune responses at day 28, except for weak correlation for NLR and IgM for moderately severe disease (r = 0.42, p <0.0489). In patients with mild illness, the levels of NLR and CRP on day 1 correlated strongly with subsequent immune responses on day 28, suggesting that this group with mild illness may represent a continuum of disease severity, with those with higher initial inflammatory markers developing immune responses more similar to those with moderate or severe illness. The levels of D-dimer on day 1 did not correlate with immune responses on day 28 in any group.

## Discussion

This is the first longitudinal study in Bangladesh to evaluate the clinical, laboratory and antibody responses in patients with differing levels of severity of illness after SARS-CoV-2 infection and also explored immune response according to disease severity. As SARS-CoV-2 is a new disease and there are many aspects regarding clinical and the immune responses that are not yet explored and remains unclear and unpredictable. Our result will facilitate to determine effective vaccination against COVID-19 and treatment of the disease. This study brought important public health information on COVID-19 in this geographical area and which may help guide for prediction of poor outcome from COVID-19. The geographical distribution of this virus’s rapid progression around the globe has unfolded a variety of clinical manifestations, outcomes and antibody responses of the disease. The clinical and immunological outcomes of COVID-19 disease and its association with various pathophysiological factors in different geographical scenario have not been studied with necessary rigor. Investigating the epidemiological characteristics with antibody responses with COVID-19 in Bangladesh will help provide a proper insight into the clinical characterization and patterns in progression of the disease. It is vital to examine these aspects and factors related to the outcomes of COVID-19 to endorse suitable means of prevention and treatment.

Bangladesh is similar to that seen globally, with age, gender and co-morbid conditions mostly associated with infections due to COVID-19 [15,16]. However, compared to other countries, the rate of death due to COVID-19 is low in Bangladesh, possibly because the demographic characteristics of our population is different, with only 10–15% of the population aged over 50 years [17].

SARS-CoV-2 infection can lead to asymptomatic or symptomatic infection, ranging from mild to severe disease and even death [18]. In our study, we found that the older and male patients suffered from symptomatic disease, which is similar to the previously reported study [15]. Patients with moderate and severe illness had more frequent cough and shortness of breath than those with mild illness; while those with mild illness had more frequent loss of taste, as seen earlier [19,20]. Most of the symptomatic patients with COVID-19 presented with fever as the first symptom, similar to patients with SARS or MERS [21]. Co-morbidities were more frequent in patients with moderate illness, and patients with more than one co-morbidity mostly had severe illness. Cardiovascular disease and diabetes known to be risk factors for susceptibility to COVID-19 infection as well as with adverse outcomes after infection [16].

Mild cases of COVID-19 had higher viral loads compared to moderate and severe cases on enrollment. Moderate and severe cases were hospitalized and enrolled at a later time point from the disease onset than mild cases. Viral load in upper respiratory specimens usually peak early in illness [22,23], and this may explain the higher viral loads from NPS at enrollment in cases with mild illness, while moderate and severe cases may have higher viral levels in lung tissue. Those with moderate and severe disease sought care in the hospital much later on after symptom onset. Age, sex and co-morbidity could also be confounders contributing to viral loads in the patients with different disease severities [24], but our small sample size did not allow us to evaluate that in this study. High viral loads in asymptomatic cases is consistent with their known role in disease transmission [22,24].

NLR, D-dimer, CRP, and ferritin were significantly higher at enrollment among patients with moderate and severe disease as reported earlier [25–28], suggesting again their importance as predictive markers for progression of disease. The NLR can be used as an early marker of disease severity, accessible to LMICs in both the hospital and outpatient setting, for prediction of severe COVID-19 case with poor outcome when other tests are not available [27].

The antibody responses and kinetics following COVID-19 of differing severity are now beginning to be elucidated [29,30]. In our study, we demonstrated that patients with moderate or severe disease had significantly higher IgM and IgG responses than mild or asymptomatic infection, although all groups had significantly higher concentrations compared to controls. The ELISA we used is based on the RBD antigen of the spike protein [1,14]. This method has previously correlated well with viral neutralization assay [31,32]. The titer of antibodies to the RBD antigen or neutralizing titer that may protect against subsequent SARS-CoV-2 infection is currently unknown [32]. Patients develop both IgM and IgG antibodies to RBD, suggesting a key role for helper T cells in isotype switching and perhaps immunologic memory after COVID-19 infection [33]. The SARS-CoV-2-specific antibodies, along with SARS-CoV-2-specific CD4+ and CD8+ T cells, have been previously reported to persist beyond 6–8 months [34]. IgG responses could be detected by day 7 after exposure to COVID-19, and peaked by the fourth week, which was similar to responses seen after SARS-CoV [35,36]. Previous data have shown that patients with severe SARS had more robust serological responses, including early seroconversion (<day 16), and higher IgG antibody levels [37], while individuals with mild SARS had lower antibody titers [38]; these results are similar to our data on SARS-CoV-2. Five individuals who died in this study from COVID-19, all had IgG or IgM levels prior to death comparable to other patients with moderate or severe disease, suggesting that there are immune factors, such as T cell responses, innate immune responses and antibody responses beyond ability of the antibody to bind to antigen, that are important in control and clearance of viral infection. It has been demonstrated that individuals with moderate-severe COVID-19 who survive, make spike-specific antibodies early in infection that are better able to engage the innate immune system than people who die [39]. In addition, individuals with mild infection have early antibody responses that are enriched for spike-specific responses compared to nucleocapsid [40] which may contribute to viral clearance in COVID-19 disease.

We found correlations between an increased level of NLR, D-dimer and CRP on study enrollment and IgM and IgG antibody titers on day 28 in those patients with mild infection, suggesting that this group may represent a continuum of severity, with those with initially higher inflammatory markers having more activation of the innate immune system more similar to those with moderate or severe infection, perhaps coupled to subsequent activation of the adaptive immune response and development of higher levels of RBD-specific antibodies. A previous study has suggested that increased neutrophil levels in early infection might lead to release of inflammatory mediators which results increased antibody responses and neutralizing antibodies to SARS-CoV-2 [41]. Interestingly, we did not find a correlation with initial levels of D-dimer and subsequent antibody levels in patients with any level of disease severity, suggesting that the D-dimer may be associated with a hypercoagulable state but not be linked to activation of the adaptive immune response. During the course of COVID-19 infection, an inflammatory reaction can change rapidly, resulting in different stages of outcomes [42]. We have shown IgM and IgG anti-SARS-CoV-2 antibodies progressively increased over different days. However, to establish an inflammatory immune response, detailed antigen specific (eg: SARS-CoV-2 peptides) T cell responses along with cytokine responses (eg: Th1, Th2, Th17 and Treg) need to be studied and is underway and will be in our future report. Primarily, we have seen higher SARS-CoV-2 specific T cell activation in severe patients compared to asymptomatic, mild and moderate patients.

Previous reports raised doubts as to the efficacy of the protection conferred by neutralizing antibodies (nAbs) in severe COVID-19 patients and suggest that enhanced nAbs might correlate with a negative clinical outcome [43,44]. We, therefore assume higher neutralizing antibody responses may dampen the inflammation in moderate and severe patients over the course of the disease. In our future studies, we will measure nAbs for better understanding of inflammation in COVID-19 patients.

There are some limitations to the study carried. First, we enrolled the participants who were able to provide consent for the study. No critical cases were enrolled who were unconscious or on life support. Secondly, the study sample size was calculated to determine the proportion of immune responses in SARS-CoV-2 confirmed patients not with the clinical parameters of COVID-19 patients. We could not measure the risk of association in different disease severity groups. In summary, we found that clinical and laboratory findings were predictive of disease severity of COVID-19. Older age, male gender, co-morbid conditions and elevated NLR, D-dimer, ferritin and CRP levels can help clinicians predict severe outcomes. Patients with moderate and severe disease developed higher levels of IgM and IgG antibodies of SARS-CoV-2 than mild or asymptomatic cases, although all infected individuals developed antibody responses higher than healthy controls.

## Supporting information

S1 Data Manuscript analytical dataset. (ZIP)

S1 STROBE Checklist Cohort studies. (DOC)
